# Probiotics prevent necrotizing enterocolitis, sepsis and mortality in preterm infants: a multicenter analysis of more than 10,000 VLBW infants in German NICUs

**DOI:** 10.1186/2047-2994-4-S1-O39

**Published:** 2015-06-16

**Authors:** LA Denkel, F Schwab, C Geffers, P Gastmeier, L Garten, B Piening

**Affiliations:** 1 Institute of Hygiene and Environmental Medicine, Charité University Medical Center Berlin, Berlin, Germany; 2 Department of Neonatology, Charité University Medical Center Berlin, Berlin, Germany

## Introduction

Enteral supplementation of probiotics has been demonstrated to reduce the risk of severe necrotizing enterocolitis (NEC) and all-cause mortality in preterm infants.

## Objectives

This retrospective analysis aimed to assess the frequency of NEC and sepsis in preterm infants with birth weights less than 1,500 grams (VLBW infants), before and after the implementation of prophylactic enteral administration of probiotics in German neonatal intensive care units (NICUs).

## Methods

This multicenter study is based on NEO-KISS, the German surveillance system for nosocomial infections in VLBW infants. All NICUs that implemented prophylactic enteral administration of probiotics with ≥ 2 strains for VLBW infants were included in the analysis. Data of VLBW infants that were admitted between 36 months before and 36 months after the implementation of probiotics were analyzed. Study period was 2004 – 2014. Interrupted time series analyses were applied to evaluate longitudinal effects of the exposition (probiotics) on the frequency of i) NEC, ii) sepsis and iii) all-cause mortality. Risk factor analyses included Cox proportional hazard regression estimating hazard ratios (HR) with 95 % confidence intervals (95 % CI).

## Results

The data of 10,890 VLBW infants - including 4,683 infants with a birth weight below 1,000 grams (ELBW infants) - from 44 neonatal departments were included in this study. Incidences of NEC and sepsis were 2.5 % (n = 274) and 15.0 % (n = 1631) in VLBW infants; 4.6 % (n = 215) and 24.2 % (n = 1133) in ELBW infants. The use of probiotics significantly reduced the risk of NEC (HR = 0.48; 95% CI = 0.39 – 0.62), all-cause mortality (HR = 0.60, 95 % CI = 0.44 – 0.83) and sepsis (HR = 0.89, 95 % CI = 0.81 – 0.98). The subgroup analysis in the ELBW infants cohort showed an even more pronounced positive effect of probiotics on NEC (HR = 0.48, 95 % CI = 0.36 – 0.64), all-cause mortality (HR = 0.59, 95 % CI = 0.41 – 0.84) and even sepsis (HR = 0.83, 95 % CI = 0.71 – 0.94).

## Conclusion

This large multicenter study provides evidence that prophylactic enteral probiotics administration significantly reduces complications of premature birth. Routine probiotic prophylaxis should be added to clinical practice as soon as possible.

## Disclosure of interest

None declared.

